# Quantifying the split-elbow sign: a comprehensive study in amyotrophic lateral sclerosis

**DOI:** 10.3389/fneur.2024.1499668

**Published:** 2024-12-03

**Authors:** Sheng-Yi He, Wei-Chen Cai, Wei-Ming Su, Qing-Qing Duan, Zheng Jiang, Kang-Fu Yin, Xiao-Jing Gu, Yong-Ping Chen, Bei Cao

**Affiliations:** ^1^Department of Neurology, West China Hospital, Sichuan University, Chengdu, China; ^2^Mental Health Center, West China Hospital, Sichuan University, Chengdu, China

**Keywords:** amyotrophic lateral sclerosis, split-elbow sign, split-elbow index, diagnosis, neuroelectrophysiology

## Abstract

**Purpose:**

The split-elbow sign (SES), characterized by preferential dysfunction of the biceps brachii compared to the triceps, is a clinical feature observed in amyotrophic lateral sclerosis (ALS). However, the quantified SES index has not been extensively investigated, and its role in diagnosing ALS remains unknown. Therefore, this study aimed to investigate the split-elbow index (SEI) derived from compound muscle action potential (CMAP), motor unit number index (MUNIX), and echo intensity (EI) in ALS.

**Methods:**

A cohort comprising 70 individuals diagnosed with ALS, along with 41 disease controls and 40 healthy controls, was recruited for the study. The SEI was calculated by dividing the recorded values of CMAP, MUNIX, and EI obtained over the biceps brachii by the corresponding value measured in the triceps, resulting in SEI_CMAP_, SEI_MUNIX_, and SEI_EI_, respectively. Receiver operating characteristic (ROC) curves of the three methods were used for comparison. Statistical analyses were performed using SPSS V.26.0 and R software.

**Results:**

Both SEI_CMAP_ and SEI_MUNIX_ exhibited significant reductions in ALS patients compared to that in controls (P_SEICMA*p*_ < 0.0001, P_SEIMUNIX_ < 0.0001), while SEI_EI_ showed an elevation (*P* < 0.0001). Furthermore, there was a notable decrease in SEI_MUNIX_ values as the disease progressed (*p* < 0.001). Moreover, ROC for SEI_MUNIX_ exhibited superior diagnostic performance (AUC = 0.846), and a comprehensive diagnostic approach combining SEI_CMAP_, SEI_MUNIX_, and SEI_EI_ resulted in AUC (0.90) on the ROC curve.

**Conclusion:**

Our study suggested that SES has emerged as a significant clinical characteristic in ALS and indicated the potential of SES indicators as biomarkers for both diagnosis and assessment of disease progression in ALS.

## 1 Introduction

Amyotrophic lateral sclerosis (ALS) is a fatal neurodegenerative disease characterized by the degeneration of upper and lower motor neurons, resulting in a wide range of motor and extra-motor system symptoms (1). The clinical presentation exhibits considerable heterogeneity among patients, with some presenting with limb muscle weakness indicative of spinal-onset disease and others manifesting dysarthria or dysphagia associated with bulbar-onset disease. Progressive muscle atrophy gradually develops in various muscle groups such as the hands, fingers, and legs (2, 3). However, this process of muscle atrophy is asymmetrical and lacks consistency in terms of speed and severity—a phenomenon referred to as dissociated muscle atrophy (4, 5). Clinically, due to the heterogeneity of ALS clinical phenotypes and a lack of special diagnostic markers in the early stages, there is an estimated delay of 14 months in diagnosing ALS (6). Therefore, this phenomenon of dissociated muscle atrophy may serve as a potential reliable diagnostic marker of ALS (7–9).

Among the observed muscle atrophy phenomena in ALS, the split-hand sign (SHS) is a well-established clinical manifestation characterized by pronounced dysfunction of the thenar group of intrinsic hand muscles, specifically the abductor pollicis brevis and first dorsal interosseous, compared to the hypothenar muscles (10–12). Furthermore, instances of the split-finger and split-leg phenomena have been documented in ALS. These clinical manifestations are characterized by more pronounced weakness and atrophy of the first flexor digitorum profundus muscle (FDP1) compared to the fourth flexor digitorum profundus muscle (FDP4) and FDP to the little finger (13), as well as heightened dysfunction of ankle plantar flexors relative to dorsiflexor muscles (2). While another study chose extensor digitorum brevis (EDB) and the abductor hallucis (AH) in ALS patients to suggest two muscles involved in the split-leg sign (14, 15).

The split-elbow sign (SES), which indicates preferential dysfunction of the biceps brachii compared to the triceps muscle, has been identified in recent studies (16, 17). Khalaf et al. observed a significant decrease in the medical research council (MRC) scores for the biceps brachii compared to those of the triceps (16). Furthermore, a study calculated the split-elbow index (SEI) using compound muscle action potential (CMAP) amplitude and demonstrated its ability to discriminate between individuals diagnosed with ALS and controls (17).

However, the assessment of the SES using MRC scores and CMAP amplitude in these studies has certain limitations. The use of MRC scores introduces subjectivity in evaluating muscle strength, potentially leading to biased results. Notably, a previous study investigating dissociated muscle atrophy of SES in ALS also used the MRC score and reported a higher score for the biceps muscle compared to the triceps muscle (18), which is inconsistent with the findings of Khalaf et al. (16).

The motor unit number index (MUNIX), a non-invasive electrophysiological measure, has demonstrated its utility as a biomarker for quantifying the functionality of lower motor neurons. In our previous study (41), we established reference values for MUNIX in five muscles, which can aid in monitoring the progression of neuromuscular diseases. Furthermore, MUNIX has been identified as a highly sensitive and effective marker for tracking the progressive loss of motor units in ALS (19–21). In addition, numerous studies also highlighted the potential of ultrasound in evaluating lower motor neuron involvement by quantifying echo intensity (EI) and fasciculation (22–24). Compared to the CMAP index alone, both MUNIX (20) and quantitative ultrasound indices, such as the echo intensity (EI) index (24), have exhibited superior sensitivity and accuracy when evaluating the severity of SHS in ALS. However, the quantified SEI derived from MUNIX and EI has not been investigated in SES. Therefore, this study aimed to investigate the characteristics of the SES using the SEI, including SEI_CMAP_, SEI_MUNIX_, and SEI_EI_ in ALS patients and explore their clinical application as biomarkers in ALS diagnosis.

## 2 Methods

### 2.1 Subjects

Patients who were diagnosed with ALS were retrospectively recruited from a well-established cohort at the ALS clinic in West China Hospital, Sichuan University, which serves as a prominent tertiary referral center in Southwestern China. The enrollment period spanned from December 2022 to July 2023. Using the revised El Escorial criteria (25), the diagnosis of ALS was meticulously established based on a comprehensive analysis of both clinical and electrophysiological findings. Ethical approval of this study was obtained from the Ethics Committee of West China Hospital of Sichuan University (2020–097). All participants signed written informed consent before enrollment.

To elucidate the characteristic of SES in ALS, we excluded patients with a history of alcohol abuse, diabetic neuropathy, carpal tunnel syndrome, and other neurological disorders. Forty patients had other neurological disorders in disease controls (DCs), including Kennedy’s disease, chronic inflammatory demyelinating polyneuropathy, multifocal motor neuropathy, facioscapulohumeral muscular dystrophy, cervical myelopathy, myasthenia gravis, and muscular dystrophy. We recruited 41 age- and gender-matched healthy volunteers who had no abnormal findings on the nerve conduction study (NCS) as healthy controls (HC).

### 2.2 Clinical information

Demographic characteristics such as age, gender, and BMI were documented for all participants. Disease duration in patients with ALS was defined as the time interval between initial symptom manifestation and screening measure evaluation. Onset patterns were classified into bulbar-onset, upper limb (UL)-onset, and lower limb (LL)-onset categories. Assessment of disease severity in each individual with ALS was conducted using the ALS Functional Rating Scale-Revised (ALSFRS-R) and King’s staging (26). Muscle strength was evaluated using the Medical Research Council (MRC) (27); a score of 0 to 4 was considered weak. The split-elbow sign presented a lower score of biceps brachii than triceps brachii.

### 2.3 Electrodiagnostic studies

The nerve conduction and MUNIX assessments were performed by the board-certified neurologist (BC) using the Viking EDX Electrodiagnostic System (Natus incorporated, United States) and a standardized methodology. Stimulation of the musculocutaneous and radial nerves was applied at the Erb’s point with supramaximal intensity, while the negative-peak CMAP amplitude over the biceps brachii and triceps brachii muscles was recorded using electrodes positioned in a belly–tendon arrangement.

For MUNIX measurements, recordings were obtained for both biceps brachii and triceps brachii muscles following the original MUNIX protocol as our previous study (41). The MUNIX was obtained using a three-step procedure according to the Natus version 22.0.2.146 (28). First, supramaximal nerve stimulation was used to record the CMAP amplitude. Then, participants activated the tested muscle by resisting the examiner in isometric contraction. The surface electromyography interference pattern was recorded when participants kept a steady contraction. Finally, participants were allowed to rest for 5–10 s before and after the maximal contraction. It was necessary to repeat the above procedure. The Viking EDX Electrodiagnostic System’s built-in software was used to automatically calculate the MUNIX values. Mean CMAP amplitude and MUNIX values were computed from the two tests. The SEI_CMAP_ and SEI_MUNIX_ were determined by dividing the recorded CMAP amplitude and MUNIX value of the biceps brachii muscle by those of the triceps brachii muscle, respectively, as follows: SEI_CMAP_ = CMAP amplitude _BICEPS BRACHII_/CMAP amplitude _TRICEPS BRACHII_, SEI_MUNIX_ = MUNIX value _BICEPS BRACHII_/MUNIX value _TRICEPS BRACHII._

### 2.4 Ultrasound examination

The neuromuscular ultrasound examinations were performed by the same physician (BC) using the same ultrasound equipment (M9 System, Mindray Bio-Medical Electronics Co., Ltd.). B-mode ultrasound was used, and all system settings remained consistent throughout the study, including parameters such as gain (56 dB), time gain compensation (in neutral position), depth (5 cm), frequency (12 MHz), compression, and focus (29). We followed the methodology outlined in a previously published study for conducting ultrasound on the biceps brachii and triceps brachii muscles (30). Participants were instructed to assume a fully relaxed supine position with their forearm in complete supination while the physician conducted ultrasound scans of their biceps brachii muscle. For triceps brachii examinations, participants were positioned in the supine position with their elbows flexed at 90 degrees and forearms resting on the abdomen. In each muscle, a region of interest (ROI) was carefully selected to encompass as much of the muscle tissue as possible, excluding any bone or surrounding fascia. The transducer was adjusted perpendicular to acquire EI values until optimal brightness was achieved.

In the data processing stage, we used the BrightnessRatio function (M9 System, Mindray Bio-Medical Electronics Co., Ltd.) for ultrasound data analysis and feature extraction. This function facilitated the personalized selection of ROI through a semi-automatic sampling box, enabling EI analysis based on gray-scale images. The gray-scale image represents the result after mapping the obtained ultrasound image, with pixel values ranging from 0 to 255 (30). The muscle EI was determined as the average pixel brightness value within the ROI. In this study, the BrightnessRatio function was used to facilitate ROI annotation for ultrasound images and automatically calculate the EI for each ROI.

The SEI_EI_ was derived by dividing the recorded EI value over the biceps brachii by that of the triceps brachii muscle, as follows: SEI_EI_ = EI_BICEPS BRACHII_ / EI_TRICEPS BRACHII._

### 2.5 Statistical analysis

Differences in demographic, electrodiagnostic, and ultrasonic data among patients with ALS, DC, and HC were analyzed. Subgroups of ALS patients were further categorized based on their ALSFRS-R score (mild ≥41, moderate 36–40, and severe ≤35) and the onset region (bulbar-onset group, upper limb-onset group, and lower limb-onset group). The Shapiro–Wilk test was used to evaluate for data normality. Differences among groups were assessed using analysis of variance (ANOVA) and the chi-square test. *Post-hoc* analysis of significant differences among groups was conducted using Tukey’s honest test. To investigate the SEI of different onset ALS, violin plots were generated. The wider portion indicated that the data were more concentrated, while the narrower portion indicated that the data were relatively small. The dotted line in the middle represented the median of the data distribution. ROC curves for SEI_CMAP_, SEI_MUNIX_, and SEI_EI_ were generated for ALS patients in comparison to non-ALS groups and produced for ALS patients compared with DCs. Statistical analyses were performed using SPSS V.26.0. software with a significance level set at a *p*-value of <0.05.

## 3 Results

In total, 70 patients who were diagnosed with ALS were enrolled in this study, consisting of 44 males and 26 females (Table 1). The mean disease duration was 14.54 ± 11.16 months, while the ALSFRS-R score averaged at 39.49 ± 6.29. King’s staging for ALS was 2.0 (1.0). In addition, a control group comprising 41 DCs and 40 HCs was recruited for comparison purposes. Age and gender distribution did not exhibit any significant differences among the ALS, DC, and HC groups (54.96 ± 10.5 vs. 52.41 ± 8.0 vs. 51.68 ± 10.74). Furthermore, based on disease severity as determined by ALSFRS-R scores, ALS patients were further categorized into three groups: “severe” (scores ≤35), “moderate” (36 ≤ scores ≤41), and “mild” (scores ≥42). Notably, it is worth mentioning that the severe group had a longer duration than both the moderate and mild groups.

**Table 1:** Comparison of demographic, electrodiagnostic, and ultrasonic findings between ALS patients, disease controls, and healthy controls^*^.

	ALS (*n* = 70)	DC (*n* = 41)	HC (*n* = 40)	*P*
Demographic profile				
Age	54.96 ± 10.5	52.41 ± 8.0	51.68 ± 10.74	0.192
Sex (female/male)	26/44	18/23	13/27	
Disease duration (M)	14.54 ± 11.16			
ALSFRS-R score	39.49 ± 6.29			
King’s staging^#^	2.0 (1.0)			
CMAP amplitude				
BB (mV)	5.40 ± 1.93^a^	6.68 ± 0.97^a^	8.3 ± 0.69^a^	0.0001
TB (mV)	8.97 ± 2.19^a^	9.57 ± 0.66^b^	11.76 ± 1.6^ab^	0.0001
SEI_CMAP_	0.61 ± 0.17^a^	0.70 ± 0.12^b^	0.72 ± 0.13^ab^	0.0001
MUNIX				
BB	85.52 ± 40.22^a^	123.80 ± 15.83^a^	177.65 ± 15.6^a^	0.0001
TB	155.78 ± 51.49^a^	178.78 ± 11.84^a^	207.75 ± 27.93^a^	0.0001
SEI_MUNIX_	0.55 ± 0.19^a^	0.69 ± 0.09^a^	0.87 ± 0.13^a^	0.0001
EI				
BB	113.80 ± 16.67^a^	85.31 ± 14.3^a^	70.44 ± 7.07^a^	0.0001
TB	84.61 ± 9.3^a^	86.51 ± 18.66^b^	62.89 ± 7.02^ab^	0.0001
SEI_EI_	1.35 ± 0.19^a^	1.02 ± 0.21^a^	1.13 ± 0.15^a^	0.0001

The values are presented as mean ± SD. *Means with the same letters are significantly different (*p* < 0.05, Tukey’s honestly significant difference test). ^a^Means *P* < 0.05 compared with each other, ^ab^ means *p* < 0.05 compared with ALS patients and DCs, respectively. ^#^King’s staging was expressed in terms of median and quartile. ALS, amyotrophic lateral sclerosis; DC, disease controls; HC, healthy controls; BB, biceps brachii; TB, triceps brachii; CMAP, compound muscle action potential; SEI split-elbow index; MUNIX, motor unit number index; EI echo intensity; M months.

Neurological disorders included in the DC group were as follows: Kennedy’s disease (2.5%), multifocal motor neuropathy (2.5%), facioscapulohumeral dystrophy (2.5%), myopathy (5.11%), myasthenia gravis (8.18%), cervical myelopathy (9.21%), and chronic inflammatory demyelinating polyradiculoneuropathy (13.35%).

**Figure 1:**
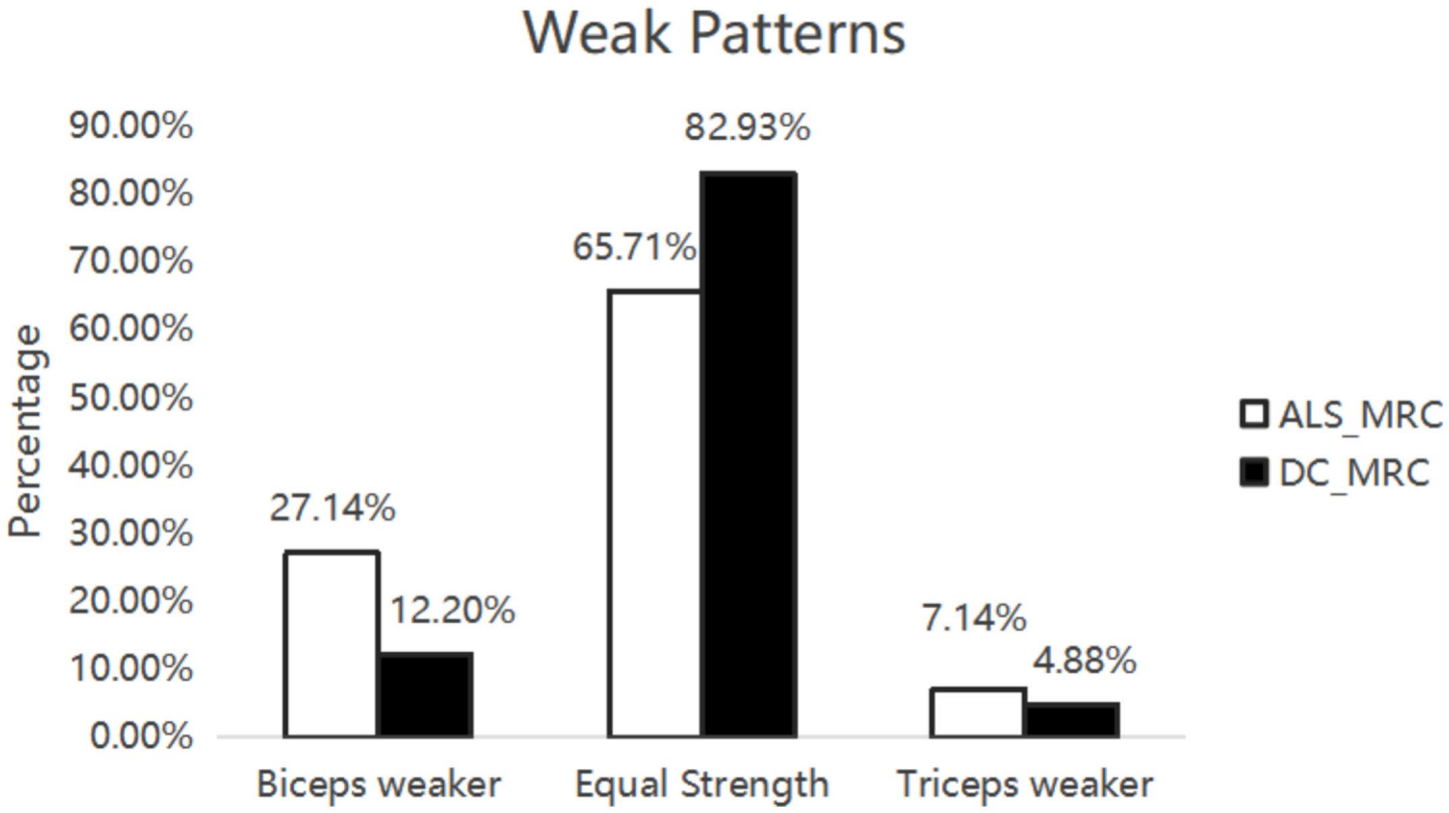
Weak patterns. Seventy ALS patients and 41 DCs were assessed muscle strength by MRC score. There was no significant difference between ALS patients and DCs.

In the ALS and DC groups, the score in MRC between biceps brachii and triceps brachii was assessed. It was not significantly different from ALS patients and DCs (X^2^ = 3.95, *p* = 0.14) (Figure 1). Biceps brachii MRC scores were significantly lower than triceps brachii MRC scores in ALS patients (*p* < 0.0001). The neurophysiological details are succinctly presented in Tables 1, 2, encompassing CMAP amplitudes, MUNIX, and EI values recorded over the biceps brachii and triceps brachii among the various groups. ALS patients exhibited significantly lower CMAP amplitudes than HCs and DCs in the biceps brachii, while such change was observed only when compared to HCs in the triceps brachii (*p* < 0.05). ALS patients exhibited significantly lower MUNIX values than HCs and DCs both in the biceps brachii and triceps brachii (p < 0.05). In addition, ALS patients exhibited significantly higher EI values than HCs and DCs in the biceps brachii, but this change was observed only when compared to HCs in the triceps brachii (*p* < 0.05). Subsequently, we calculated SEI to quantify the degree of preferential weakness and atrophy in muscles. The SEI_MUNIX_ and SEI_CMAP_ were found to be significantly lower in ALS patients than those in DCs and HCs, and the SEI_EI_ was significantly higher than that in DCs and HCs (Table 1) (*p* < 0.05).

**Table 2:** Comparison of clinical, electrodiagnostic, and ultrasonic findings among ALS, disease controls, and healthy controls according to disease severity using the ALSFRS-R^*^.

	HC (*n* = 40)	DC (*n* = 41)	Mild (*n* = 30)	Moderate (*n* = 27)	Severe (*n* = 13)	*P*
Demographic profile						
Age	51.67 ± 10.74	52.41 ± 8.00	53.13 ± 9.86	56.07 ± 10.53	56.85 ± 11.69	0.28
Sex (female/male)	13/27	18/23	11/19	13/14	3/10	0.05
Disease duration (months)			12.71 ± 8.62	13.22 ± 8.68	21.54 ± 17.54	0.04
CMAP amplitude						
BB (mV)	8.30 ± 0.69	6.68 ± 0.97^a^	5.74 ± 1.67^a^	5.58 ± 2.00^ab^	4.24 ± 2.05^abc^	0.001
TB (mV)	11.76 ± 1.56	9.57 ± 0.66^a^	9.24 ± 1.96^a^	9.10 ± 2.07^a^	8.05 ± 2.81^a^	0.001
SEI_CMAP_	0.72 ± 0.13	0.70 ± 0.13	0.63 ± 0.16	0.62 ± 0.17	0.53 ± 0.18^ab^	0.001
MUNIX						
BB	177.65 ± 15.60	123.80 ± 15.83^a^	104.87 ± 42.30^ab^	80.63 ± 28.70^abc^	51.08 ± 30.29^abcd^	0.001
TB	207.75 ± 27.94	178.78 ± 11.85^a^	172.77 ± 60.34^a^	154.96 ± 40.86^a^	118.31 ± 24.25^abcd^	0.001
SEI_MUNIX_	0.87 ± 0.13	0.69 ± 0.09^a^	0.61 ± 0.16^a^	0.53 ± 0.18^ab^	0.43 ± 0.23^abc^	0.001
EI						
BB	70.44 ± 7.07	85.31 ± 14.30^a^	103.38 ± 15.23^ab^	115.56 ± 15.52^abc^	121.13 ± 22.72^abc^	0.001
TB	62.88 ± 7.02	86.51 ± 18.66^a^	81.31 ± 8.09^a^	88.53 ± 10.54^a^	84.36 ± 8.34^a^	0.001
SEI_EI_	1.13 ± 0.15	1.02 ± 0.22	1.27 ± 0.15^ab^	1.32 ± 0.22^ab^	1.44 ± 0.27^ab^	0.001

The values are presented as mean ± SD. ^*^Means with the same letters are significantly different (Tukey’s honestly significant difference test). ^a^Means *p* < 0.05 compared with HC, ^b^ means *p* < 0.05 compared with DC, ^c^ means p < 0.05 compared with the mild ALS group, and ^d^ means *p* < 0.05 compared with the moderate group. ALS, amyotrophic lateral sclerosis; DC, disease controls; HC, healthy controls; BB, biceps brachii; TB, triceps brachii; CMAP, compound muscle action potential; SEI split-elbow index; MUNIX, motor unit number index; EI echo intensity.

**Figure 2:**
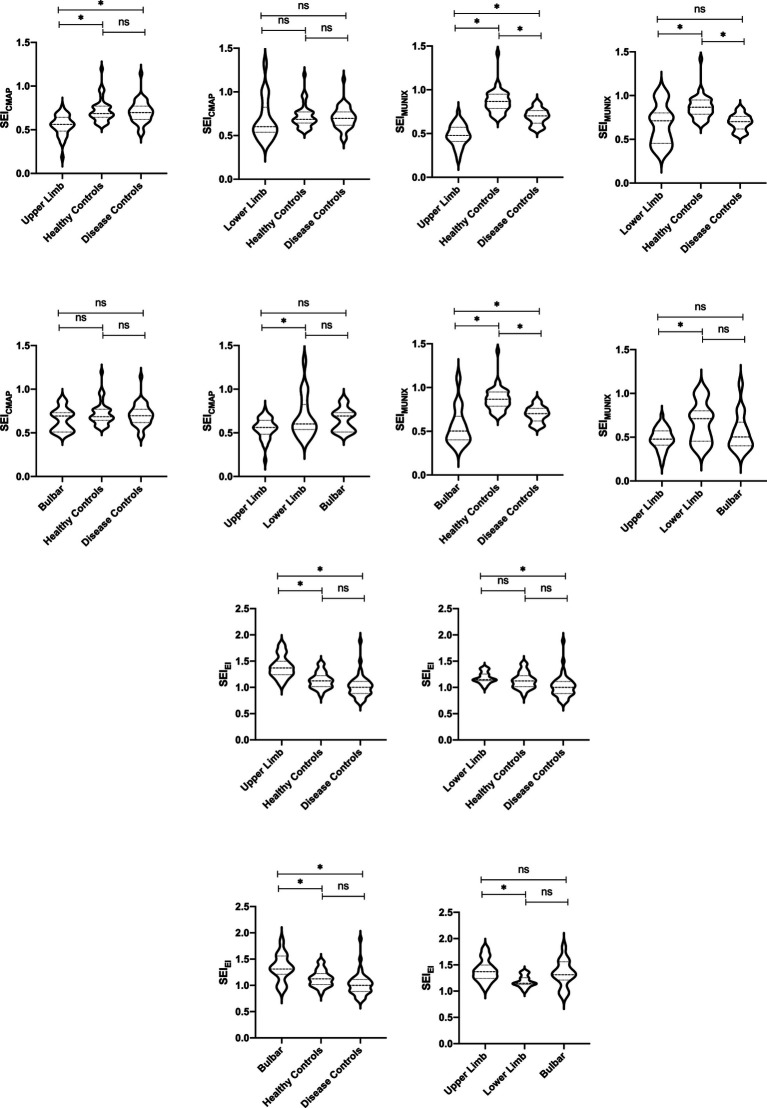
CMAP amplitude and SEI_CMAP_ among different ALS onset, DCs, and HCs. *p < 0.05. ns: non-significant **(A)**. MUNIX value and SEI_MUNIX_ among different ALS onset, DCs, and HCs. *p < 0.05. ns: non-significant **(B)**. EI value and SEI_EI_ among different ALS onset, DCs, and HCs. *p < 0.05. ns: non-significant **(C)**.

The subgroup analysis revealed a significantly lower level of SEI_MUNIX_ in different severity groups with ALS compared to DCs and HCs. Furthermore, SEI_MUNIX_ values decreased as the disease progressed, according to the ALSFRS-R score (*p* < 0.001). However, no significant differences were observed in SEI_CAMP_ and SEI_EI_ among different severity groups with ALS (*p* > 0.05) (Table 2). By comparing the median of the different violin plots, we could preliminarily determine whether there was a difference in the central trend between the different groups. Overall, the upper limb-onset group exhibited the lowest SEI_CMAP_ and SEI_MUNIX_ values, while the upper limb-onset group had the highest SEI_EI_ values than lower limb- and bulbar-onset groups (Figures 2A–C) (*p* < 0.05).

**Figure 3:**
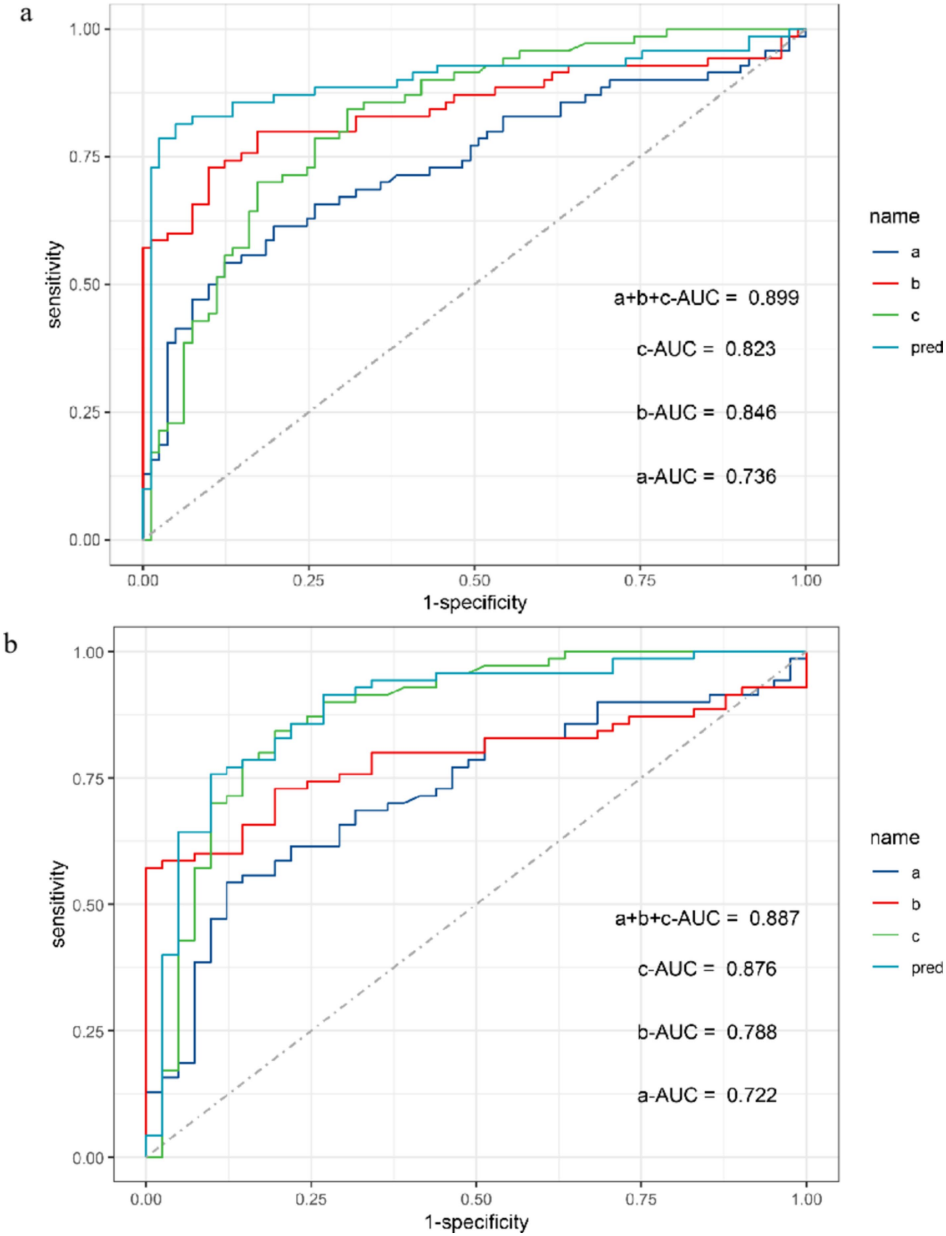
ROC curve for SEI_CMAP_, SEI_MUNIX_, SEI_EI_, and combined three methods between ALS and non-ALS groups **(A)**. ROC curve for SEI_CMAP_, SEI_MUNIX_, SEI_EI_, and combined three methods between ALS and DCs groups **(B)**. a: SEI_CMAP_, b: SEI_MUNIX_, c: SEI_EI._ Sensitivity refers to the ability of a diagnostic test to correctly diagnose an actually sick person as a patient and specificity refers to the ability of a diagnostic test to correctly diagnose an actually disease-free person as a non-patient. As the “1-specificity” increases (specificity decreases), the sensitivity tends to be faster and then slower until it flattens out. The area under the curve (AUC) for SEI_CMAP_, SEI_MUNIX_, and SEI_EI_ is 0.736, 0.846, and 0.823, respectively **(A)**. The AUC for SEI_CMAP_, SEI_MUNIX_, and SEI_EI_ is 0.722, 0.788, and 0.876, respectively **(B)**.

The ROC curve was used to conduct power analysis for SEI_CMAP_, SEI_MUNIX_, and SEI_EI_. The area under curve (AUC) of SEI_CMAP_ was 0.74 (95% CI: 0.65–0.82), with a cut-off value of 58.7 distinguishing ALS patients from controls with a sensitivity of 87.7% and specificity of 54.3%. For SEI_MUNIX_, the AUC was 0.85 (95% CI: 0.78–0.92), and the cut-off value of 60.6 differentiated ALS patients from controls with a sensitivity of 90.1% and specificity of 72.9%. As for SEI_EI_, the AUC was determined as 0.82 (95% CI: 0.76–0.89), while the cut-off value of 113 differentiated ALS patients from controls with a sensitivity of 69.1% and specificity of 84.3% (Supplementary Figures S1a–d). In addition, we conducted diagnostic power comparisons among SEI_CMAP_, SEI_MUNIX_, and SEI_EI_ using R software package. The results revealed that there was a significant difference between SEI_CMAP_ and SEI_MUNIX_ (*p* < 0.05), indicating that SEI_MUNIX_ exhibited diagnostic power. However, the comparison between these curves for SEI_MUNIX_ and SEI_EI_ did not yield significant differences (*p* = 0.613). Furthermore, a comprehensive diagnosis strategy combining these methods resulted in an ROC curve with an AUC value of 0.90 (95% CI 0.84–0.96), sensitivity of 95%, and specificity of 81% (Figure 3A). SEI_EI_ might present better power for diagnosis in ALS patients and DCs (AUC 0.876, *p* = 0.012), while the cut-off value of 113 could differentiate ALS patients from controls with a sensitivity of 80.5% and specificity of 84.3% (Figure 3B).

## 4 Discussion

Collectively, we conducted a cohort study to validate SES as a clinical feature in ALS and establish a comprehensive diagnostic approach. When compared to DCs and HCs, the SEI_EI_ increased, while SEI_CAMP_ and SEI_MUNIX_ decreased significantly in ALS patients, indicating quantified weakness of biceps brachii relative to triceps brachii using SEI measurements. In addition, we observed progressively lower SEI_MUNIX_ values in ALS patients with advancing disease stages defined by the ALSFRS-R. To assess diagnostic accuracy, an ROC curve was drawn to determine which method was more suitable for diagnosing ALS. Overall, this study provides support for the presence of dissociated muscle atrophy in ALS through electrodiagnostic and ultrasound methods combined with clinical diagnosis and suggest that SEI_MUNIX_ could serve as a predictor of early diagnosis and disease progression. SEI_EI_ might be helpful to distinguish ALS from other neurological disorders.

Prior to discussing the presence of the split-elbow sign and the utility of the SEI, it is important to note that the split-hand phenomenon was first identified and proposed (31, 32). SHS likely represents an early and highly specific clinical manifestation of ALS (7, 33). Regarding potential pathophysiological mechanisms, cortical motoneuronal hyperexcitability appears to be the most plausible cause of dissociated muscle atrophy (34). In addition, an another study suggested that the intrinsic hand muscles occupy a more prominent cortical somatotopic location, which may contribute to glutamate-mediated hyperexcitability through larger cortical projection neurons (35). As for SES, previous studies have consistently demonstrated that the triceps brachii muscle is relatively preserved compared with the biceps brachii muscle, as confirmed by various methodologies (16, 17). Transcranial magnetic stimulation experiments have revealed a higher influx of corticomotoneuronal inputs to the biceps brachii than to the triceps brachii (36). These suggest that corticomotoneuronal hyperexcitability may also contribute to SES. However, a study conducted in Asia reported inconsistent results for SES and indicated preferential weakness in elbow extensors over flexors (18). In contrast, our study found that biceps brachii MRC scores were significantly lower than triceps brachii MRC scores in ALS patients. In addition, biceps brachii MRC scores were not significantly lower in ALS patients than in DCs in our study, which is consistent with the study by Pavey et al. (17). This may be related to disease severity of ALS, and there is a certain subjectivity when using this assessment tool. Hence, our study applies electrodiagnostic and ultrasound examinations to reaffirm that SES is indeed a clinical feature of ALS. Our study further supports the notion of preferential dysfunction in the biceps brachii muscle. Considering that subjective estimation may introduce potential bias into MRC muscle score system assessments, it implies that more precise techniques are required alongside this scoring system.

To quantify the SHS as a potential diagnostic biomarker of ALS, the split-hand index was established (8). In line with this, the SEI, which is derived neurophysiologically from CAMP amplitudes and the MUNIX values recorded by the biceps and triceps brachii, may also serve as a novel diagnostic biomarker in ALS (37). However, it should be noted that compensatory collateral reinnervation by remaining motor neurons can potentially influence CMAP amplitude in denervated muscle fibers (24). Ultrasound imaging remains an effective method for measuring EI due to its minimal interference from compensatory reinnervation processes (38), thus suggesting an advantage for SEI_EI_ based on these considerations. Our study used SEI_CAMP_, SEI_MUNIX_, and SEI_EI_ measurements to find statistically significant differences in SEI between ALS patients and controls.

To assess the efficacy of the three methods in different stages of ALS, we categorized ALS patients into mild, moderate, and severe groups according to their ALSFRS-R scores. SEI_MUNIX_ revealed a significant distinction when compared among ALS,DCs, and HCs. Although the difference between HCs and DCs was significant, it was inevitable because DCs included patients with similar symptoms but not ALS. Therefore, the significant difference between the DC and ALS groups was more concerning. More importantly, a progressive decline in SEI_MUNIX_ values was observed as ALS patients progressed through the disease stages. In line with this finding, Kim et al. also reported a significant correlation between split-hand index MUNIX (SI_MUNIX_) and ALSFRS-R (*r* = 0.33) (20). Hence, these results suggested that SEI_MUNIX_ could serve as an early diagnostic marker and an indicator of disease progression. While SEI_EI_ exhibited significantly higher values than controls at each stage of ALS, there were no notable differences in SEI_EI_ and SEI_CMAP_ among subgroups of different severity within the ALS population; indicating that these two measures may hold promise as differential diagnostic indices but are not suitable for assessing disease progression. However, further studies with larger sample sizes are warranted to validate these findings. Interestingly, SEI_CAMP_ and SEI_MUNIX_ have lower statistical significance in the upper limb-onset ALS patients, but SEI_EI_ is higher in the upper limb-onset group, which is consistent with the results of SHS by EI (24, 39). This alignment can be attributed to the fact that EI evaluates alterations in the muscular architecture and infiltrated muscular fat and fiber content (30).

In our study, we used the ROC curves for SEI_CAMP_, SEI_MUNIX_, and SEI_EI_ and compared the AUC for diagnostic power of the above methods. We found that SEI_MUNIX_ had significantly better performance than SEI_CAMP_, but there was no significant difference between SEI_MUNIX_ and SEI_EI_. Neuwirth et al. also found MUNIX as an electrophysiological marker was significantly better than CMAP for testing lower motor neuron loss (40). It was consistent that MUNIX and SEI_MUNIX_  could be better potential markers than CMAP and SEI_CAMP_. In addition, the ROC curve was used for three methods between the ALS group and DCs. There was a significant difference in SEI_EI_ compared to SEI_CMAP_. A previous study found that EI and fasciculation quantified by muscle ultrasound were tested for sensitivity and specificity to distinguish ALS and non-ALS (39). Hence, for the efficiency verification of two methods, it might take more participants to observe whether there is a difference between SEI_MUNIX_ and SEI_EI_. In addition, considering that the diagnosis of ALS requires a combination of clinical symptoms and electrophysiological strategies, we combined the three methods as a joint diagnostic strategy and compared their diagnostic accuracy and found that it seemed to be more significantly helpful as a biomarker.

Of course, some potential limitations in this study should be considered: First, the small number of ALS patients and controls is a possible limitation. Then, since our study was a retrospective cross-sectional design, it is necessary to use SEI_MUNIX_ and SEI_EI_ tracking ALS progression as a longitudinal study in the future. Third, the MUNIX depends on whether patients can complete the corresponding movements of the target muscles; hence, patients with poor strength or fit would bias the results. Further studies with larger, multi-center, disease control and prospective design are needed to clarify the diagnostic utility of SEI_MUNIX_ and SEI_EI_ in ALS.

## 5 Conclusion

The SES is significantly more common in ALS patients when compared to disease controls and healthy controls. It could be supported by the reduction of SEI_MUNIX_ and the elevation of the SEI_EI_. Importantly, our study found that SEI_MUNIX_ could serve as an early diagnostic marker and an indicator of disease progression. Although the ROC of the SEI_MUNIX_ indicates that it is a great approach for the diagnosis of ALS, the combined methods are important for diagnosis.

## Data Availability

The raw data supporting the conclusions of this article will be made available by the authors, without undue reservation.

## Supplementary material

Supplementary figure 1 Between ALS and non-ALS groups, the area under the curve (AUC) for SEICMAP, SEIMUNIX, and SEIEI is 0.736, 0.846, and 0.823, respectively **(a-c)**. The AUC for SEIEI is 0.876 between ALS and DCs **(d)**.
