# Supplementary figures and images for: Quantifying the split-elbow sign: a comprehensive study in amyotrophic lateral sclerosis
Source: Front Neurol. 2024 Dec 3;15:1499668. doi: 10.3389/fneur.2024.1499668 (PMC11653355; doi:10.3389/fneur.2024.1499668)

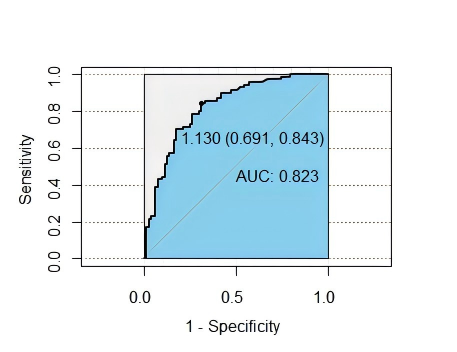

Supplement: Supplementary figure 1 — Between ALS and non-ALS groups, the area under the curve (AUC) for SEICMAP, SEIMUNIX, and SEIEI is 0.736, 0.846, and 0.823, respectively (a-c). The AUC for SEIEI is 0.876 between ALS and DCs (d). [file Image_1.tif]

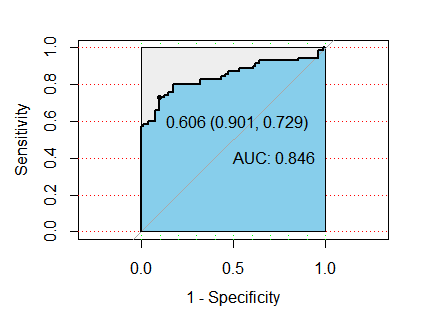

Supplement: Supplementary file 2 [file Image_2.tif]

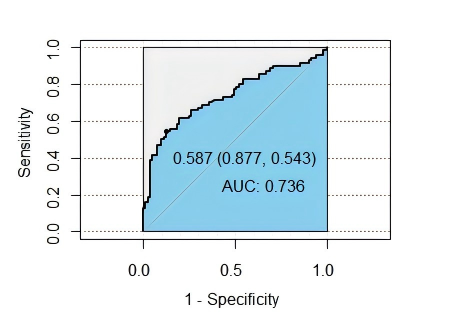

Supplement: Supplementary file 3 [file Image_3.tif]

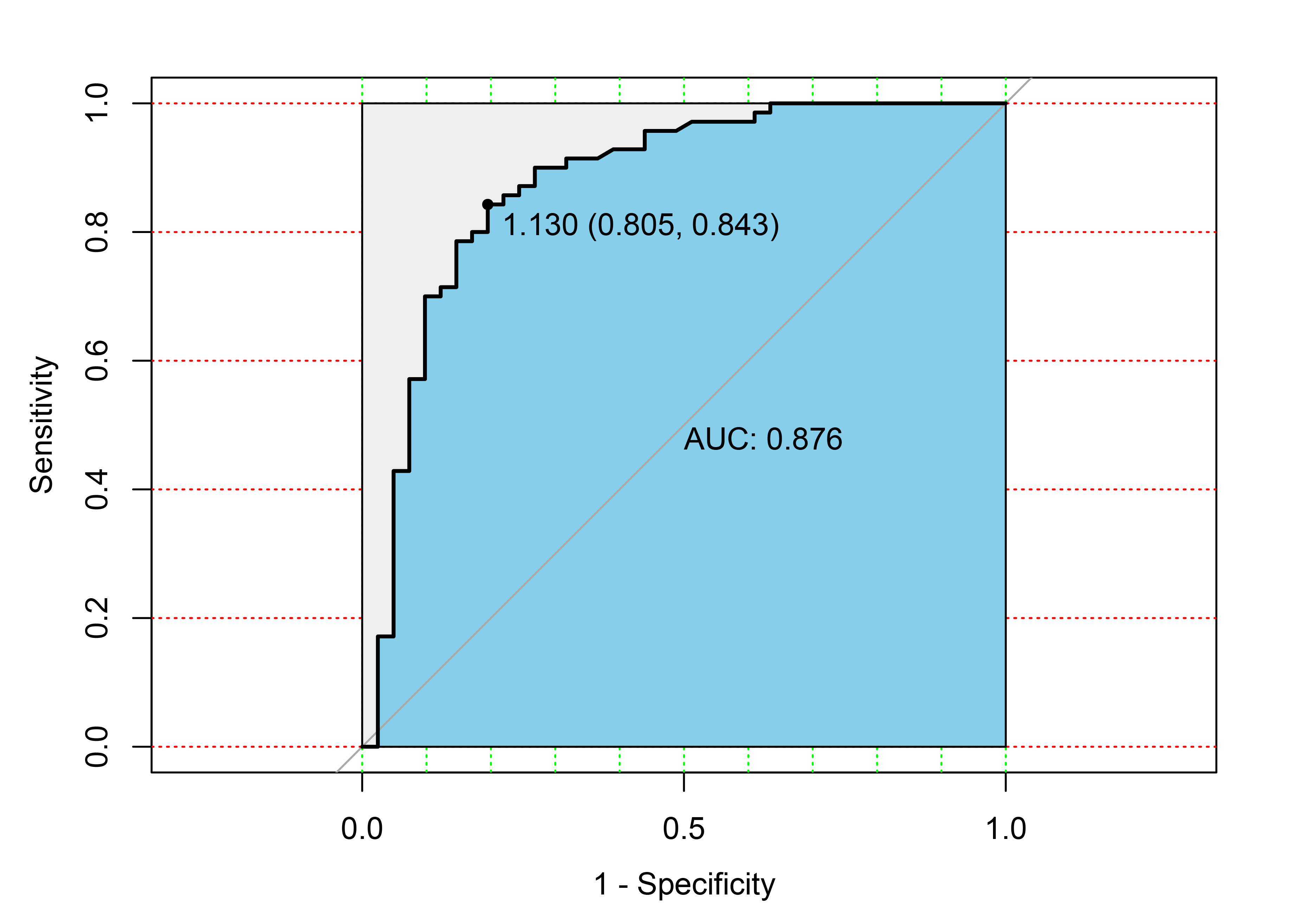

Supplement: Supplementary file 4 [file Image_4.tif]
